# Life-cycle energy use and greenhouse gas emissions of production of bioethanol from sorghum in the United States

**DOI:** 10.1186/1754-6834-6-141

**Published:** 2013-10-02

**Authors:** Hao Cai, Jennifer B Dunn, Zhichao Wang, Jeongwoo Han, Michael Q Wang

**Affiliations:** 1 Systems Assessment Group, Energy Systems Division, Argonne National Laboratory, 9700 South Cass Avenue, Argonne, IL 60439, USA

**Keywords:** Grain sorghum, Sweet sorghum, Forage sorghum, Ethanol, Life-cycle analysis, Greenhouse gas emissions

## Abstract

**Background:**

The availability of feedstock options is a key to meeting the volumetric requirement of 136.3 billion liters of renewable fuels per year beginning in 2022, as required in the US 2007 Energy Independence and Security Act. Life-cycle greenhouse gas (GHG) emissions of sorghum-based ethanol need to be assessed for sorghum to play a role in meeting that requirement.

**Results:**

Multiple sorghum-based ethanol production pathways show diverse well-to-wheels (WTW) energy use and GHG emissions due to differences in energy use and fertilizer use intensity associated with sorghum growth and differences in the ethanol conversion processes. All sorghum-based ethanol pathways can achieve significant fossil energy savings. Relative to GHG emissions from conventional gasoline, grain sorghum-based ethanol can reduce WTW GHG emissions by 35% or 23%, respectively, when wet or dried distillers grains with solubles (DGS) is the co-product and fossil natural gas (FNG) is consumed as the process fuel. The reduction increased to 56% or 55%, respectively, for wet or dried DGS co-production when renewable natural gas (RNG) from anaerobic digestion of animal waste is used as the process fuel. These results do not include land-use change (LUC) GHG emissions, which we take as negligible. If LUC GHG emissions for grain sorghum ethanol as estimated by the US Environmental Protection Agency (EPA) are included (26 g CO_2_e/MJ), these reductions when wet DGS is co-produced decrease to 7% or 29% when FNG or RNG is used as the process fuel. Sweet sorghum-based ethanol can reduce GHG emissions by 71% or 72% without or with use of co-produced vinasse as farm fertilizer, respectively, in ethanol plants using only sugar juice to produce ethanol. If both sugar and cellulosic bagasse were used in the future for ethanol production, an ethanol plant with a combined heat and power (CHP) system that supplies all process energy can achieve a GHG emission reduction of 70% or 72%, respectively, without or with vinasse fertigation. Forage sorghum-based ethanol can achieve a 49% WTW GHG emission reduction when ethanol plants meet process energy demands with CHP. In the case of forage sorghum and an integrated sweet sorghum pathway, the use of a portion of feedstock to fuel CHP systems significantly reduces fossil fuel consumption and GHG emissions.

**Conclusions:**

This study provides new insight into life-cycle energy use and GHG emissions of multiple sorghum-based ethanol production pathways in the US. Our results show that adding sorghum feedstocks to the existing options for ethanol production could help in meeting the requirements for volumes of renewable, advanced and cellulosic bioethanol production in the US required by the EPA’s Renewable Fuel Standard program.

## Background

Biofuels have been promoted in the US and other countries for reducing greenhouse gas (GHG) emissions and petroleum fuel consumption. The US 2007 Energy Independence and Security Act (EISA) administers the Renewable Fuel Standard (RFS) program with a total volumetric requirement of 136.3 billion liters of renewable biofuels by 2022 [1]. This total includes 56.8 billion liters of renewable biofuel, 18.9 billion liters of advanced biofuel, and 60.6 billion liters of cellulosic biofuel, which have a life-cycle GHG emission reduction by at least 20%, 50%, and 60%, respectively, relative to gasoline [2]. Bioethanol is now the dominant biofuel used in the transportation sector. The current US bioethanol industry has been developed to use high starch content feedstocks, primarily corn, to produce ethanol. To meet the EISA volumetric requirement, a variety of feedstocks may be used including other starch- and sugar-based crops, as well as crop residues (corn stover, wheat straw, rice straw, and sugarcane straw, among others), dedicated energy crops (e.g., switchgrass, miscanthus, mixed prairie grasses, and short-rotation trees), and forest residues.

The three varieties of sorghum (grain, sweet, and forage) have recently received increased attention as biofuel feedstocks. Drought-tolerant grain sorghum (GS), which produces seedheads that are typically harvested for livestock feed, is the dominant sorghum type. It is mostly grown in the central United States (Kansas, Colorado, Nebraska, Missouri, and South Dakota) and the southern plains (Texas and Oklahoma), totaling 2.53 million hectares in 2012 [3]. Sweet sorghum (SS) is a tropical grass of African origin [4]. SS stores non-structural carbohydrates in its stem and does not produce a large seed head [5]. It is found primarily in the south-central and south-eastern United States, and is grown for syrup on small scale with 553 hectares of SS harvested in 2007 [6]. SS has emerged as a potential feedstock candidate for bioethanol production because of its low water demand, short growing period, and sugar and biomass yield potential in less desirable conditions such as semi-arid and salty lands [7]. SS is harvested at a different time of the year than sugarcane, but can be processed for bioethanol using similar, if not identical, equipment [8]. Forage sorghum (FS) is grown on 147 thousand hectares in the US in 2012 with a total production of 2.1 million dry tonnes of cellulosic biomass [9]. This type of sorghum can be grown under conditions that are unfavorable for corn production, has high biomass yield [10], and has attracted interest as a potential bioenergy crop for cellulosic ethanol production [11].

Thirty to thirty-five percent of the total GS produced in the US is devoted to ethanol production [12,13], which is eqaul to about 1.1–1.2 billion liters ethanol, or about 2% of the US ethanol production. GS was co-fed with corn by six US ethanol plants in 2012 [14]. POET’s South Dakota ethanol plant, the second-largest in the US, will begin to use GS as a co-feedstock with corn [15]. Further, there is growing interest in cultivating SS as an ethanol feedstock. For example, SS ethanol plants are being considered in Florida [16].

A handful of life-cycle analyses (LCA) of sorghum ethanol have been conducted. Wang et al. [17] conducted an LCA of US GS ethanol. They reported a positive net energy benefit of 7.11 MJ per liter GS ethanol. Most recently, the US Environmental Protection Agency (EPA) published the final rule approving GS fuel pathways [18]. EPA’s analysis indicates that GS ethanol produced at dry-mill facilities using fossil natural gas (FNG) for process energy meets the GHG emissions reduction threshold of 20% relative to baseline petroleum fuel, thus qualifying as a renewable fuel under the RFS. The rule also specifies that GS ethanol produced at dry-mill facilities that use biogas from landfills, animal waste, or waste treatment for process energy meets a GHG emission reduction of at least 50% relative to petroleum gasoline, thus qualifying as an advanced biofuel. The EPA’s rule and analysis target GS as the feedstock for bioethanol production. Despite recognition of SS and FS as possible ethanol feedstocks, LCAs of dedicated ethanol production pathways for SS and FS as advanced biofuels feedstocks have yet to be conducted.

To examine the life-cycle energy use and GHG emissions of sorghum-based ethanol production, we conducted a well-to-wheels (WTW) analysis of the energy use and GHG emission impacts of sorghum-based ethanol production in the US using GS, SS, and FS as feedstocks. The analysis includes an assessment of uncertainty in sorghum ethanol LCA through incorporation of probability distribution functions (PDFs) of key parameters developed from an extensive review of the literature. For this study, we expanded and updated the GREET™ (Greenhouse gases, Regulated Emissions, and Energy use in Transportation) model, which we have developed at Argonne National Laboratory [19], to include the GS-, SS-, and FS-based ethanol pathways. GREET is a widely-used model that systematically examines the life-cycle energy use and emissions associated with many conventional and advanced vehicle and fuel technologies.

## Results

**Figure 1:**
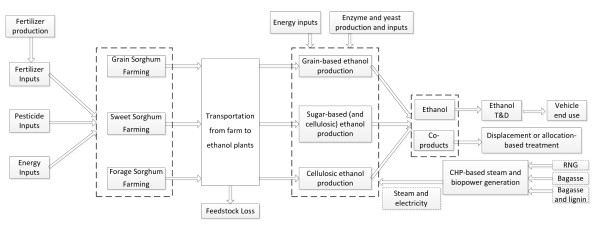
System boundary of well-to-wheels analysis of sorghum-based ethanol.

Figure 1 presents the system boundary for the WTW analysis of the five sorghum-based bioethanol pathways described in Table 1 that were included in our analysis. These five pathways differ in feedstock, farming practice, conversion technologies, process fuel supply, and co-product handling methods. The baseline petroleum gasoline pathway is also analyzed for comparison. The functional unit we use is MJ of ethanol or gasoline.

**Table 1:** Five sorghum-based ethanol production pathways and scenarios

**Pathway**	**Ethanol feedstock**	**Process fuel supply**	**Vinasse fertigation**	**CHP**^**a **^**feedstock**	**Co-product**	**Co-product handling methods**
**I**	Grain, GS	FNG, electricity		None	WDGS^b^	Displacement
**II**	Grain, GS	RNG^c^ from AD^d^ of animal waste		RNG	WDGS	Displacement
**III(a)**	Sugar, SS	Steam and electricity from CHP facilities	No	Sorghum bagasse	Grain and electricity	Displacement for grain (animal feed), energy-based allocation for electricity^e^
**III(b)**	Sugar, SS	Steam and electricity from CHP facilities	Yes	Sorghum bagasse	Grain, vinasse and electricity	Displacement for grain (animal feed) and vinasse (fertilizer), energy-based allocation for electricity
**IV**	Cellulosic biomass, FS	Steam and electricity from CHP facilities		Lignin residue and a portion of cellulosic biomass	Electricity	Energy-based allocation
**V(a)**	Sugar and bagasse, SS	Steam and electricity from CHP facilities	No	Lignin residue and a portion of the bagasse	Grain and electricity	Displacement for grain (animal feed) and energy-based allocation for electricity
**V(b)**	Sugar and bagasse, SS	Steam and electricity from CHP facilities	Yes	Lignin residue and a portion of the bagasse and	Grain, vinasse and electricity	Displacement for grain (animal feed) and vinasse (fertilizer) and energy-based allocation for electricity

^a^ Combined heat and power;

^b^ Wet distillers grains with solubles. The current grain sorghum ethanol industry produces an average of 92% WDGS [18];

^c^ Renewable natural gas. We assume that the animal waste is transported by trucks from the farms to the AD plants, and the RNG produced at the AD plants is assumed to be transported to the ethanol plants via pipeline;

^d^ Anaerobic digestion;

^e^ The displacement method is used to handle SS grain, because it, instead of being an energy product like ethanol or electricity, can be used as animal feed like corn grain. The energy-based allocation is used for electricity because it is an energy product like ethanol.

Pathway I and Pathway II simulate starch-to-ethanol production from GS grains at a dry-mill ethanol plant, using FNG and renewable natural gas (RNG) from anaerobic digestion (AD) of animal waste, respectively, as process fuel. RNG is fed to a combined heat and power (CHP) system to co-generate steam and electricity to meet process demands in Pathway II. Pathway III examines SS ethanol production from the fermentable sugar extracted from the sucrose-rich SS stalk. A CHP system using bagasse as the feedstock permits co-generation of sufficient steam and electricity to meet process demands. For this pathway, we also assessed the impacts of applying co-produced vinasse, the ethanol distillation effluent rich in potassium, nitrogen, and other minerals, to soil thereby offsetting some conventional fertilizer use. Pathway IV examines cellulosic ethanol production using FS as the feedstock. The lignin residue and part of the FS feedstock are also diverted to the CHP system so that process steam and electricity demand are met without consumption of supplemental energy. Pathway V simulates both sugar-based and cellulosic ethanol production, using the extracted sugar and some of the bagasse of SS as the feedstock for ethanol production. The lignin residue and the balance of the bagasse are fed to the CHP system, which again generates enough steam and electricity to meet process demands. Two scenarios of Pathway V listed in Table 1 are assessed.

### Well-to-wheels energy use

**Figure 2:**
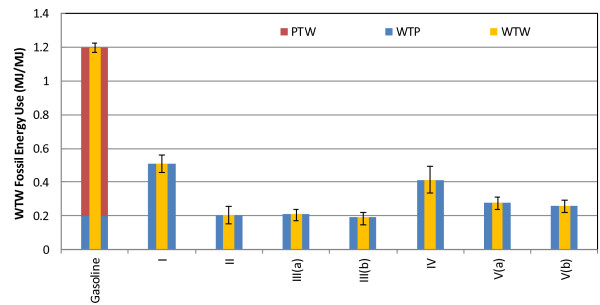
Well-to-wheels fossil energy use (MJ/MJ) of sorghum-based bioethanol, in comparison to gasoline.

Our analysis focused on WTW results for energy use and GHG emissions. We present the WTW fossil energy use results, divided into well-to-pump (WTP) and pump-to-wheels (PTW) phases, in Figure 2. The lower and upper error bars represent the 10th percentile (P10) and the 90th percentile (P90), respectively, of the stochastic simulations of the WTW results. The results for total energy use, which include fossil (petroleum, natural gas, and coal) and renewable fuel use, are presented in Additional file 1.

In Figure 2, all ethanol pathways consume less fossil energy to produce ethanol than the product fuel contains. Pathways IV, V(a), and V(b) exhibit significantly lower fossil fuel consumption because a portion of the feedstock is fed to a CHP system, producing sufficient heat and power to meet process energy demands. Similarly, CHP reduces fossil energy demand in Pathway III(b). This pathway has the lowest fossil energy consumption because excess electricity from the CHP is exported to the grid and co-produced vinasse offsets some conventional fertilizer. GS is competitive with the SS and FS pathways when RNG is the fuel for the conversion step. Fossil energy consumption more than doubles for GS when FNG is the conversion-step process fuel. Relative to gasoline, sorghum-based ethanol can achieve a reduction, on average, between 57% and 84%, as shown in Table 2.

**Table 2:** Reductions (%) of WTW fossil energy use and GHG emissions by sorghum-based ethanol, relative to gasoline

		**I**	**II**	**III(a)**	**III(b)**	**IV**	**V(a)**	**V(b)**
Fossil energy	Mean	57	83	82	84	66	77	78
	Range^a^	52–63	78–88	80–86	81–88	58–73	74–81	75–82
GHG	Mean	35	56	71	72	49	70	72
	Range^a^	−3–60	18–83	55–82	57–84	28–65	57–80	58–82

^a^ The lower bound of the range is the ratio of the difference of the P10 value of the baseline gasoline and the P90 value of each pathway to the P10 value of gasoline. The upper bound of the range is the ratio of the difference of the P90 value of gasoline and the P10 value of each pathway to the P90 value of gasoline.

Energy balances and energy ratios, the difference or ratio of the energy content of ethanol and the fossil energy used to produce it [20], respectively, are presented in Table 3. All bioethanol production scenarios have positive energy balances and energy ratios greater than one. Our estimated energy balance of GS-based ethanol production agrees with that of Wang *et al*. [17].

**Table 3:** Energy balance and energy ratio of sorghum ethanol pathways

	**I**	**II**	**III(a)**	**III(b)**	**IV**	**V(a)**	**V(b)**
Energy balance, MJ^a^/Liter	10.4	17.0	16.8	17.2	12.5	15.4	15.7
Energy ratio	2.0	4.9	4.7	5.2	2.4	3.6	3.8

^a^ Lower heating value.

### WTW GHG emissions

**Figure 3:**
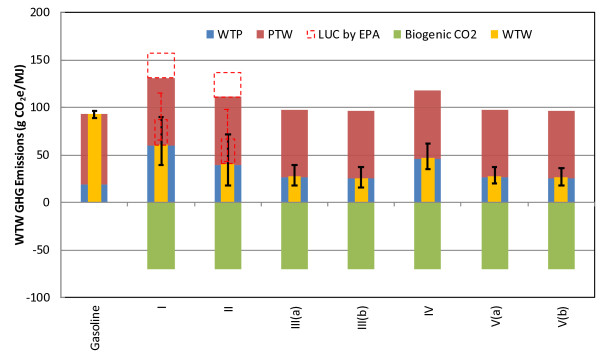
**Well-to-wheels GHG emissions (g CO**_**2**_**e/MJ) of sorghum-based ethanol pathways, in comparison to baseline gasoline.** Error bars in red represent the results of GS-based ethanol when the LUC GHG emissions (26 g CO_2_e/MJ) estimated by EPA are included.

Figure 3 shows WTW GHG emissions of the sorghum-based bioethanol pathways. Gasoline here is gasoline blending stock without ethanol or other oxygenates. WTW GHG emissions are separated into WTP, PTW, and biogenic CO_2_ (i.e., carbon in bioethanol) emissions. Combustion emissions are the largest GHG emission source for all fuel pathways. However, in the bioethanol cases, the uptake of CO_2_ during feedstock production almost entirely offsets ethanol combustion GHG emissions. Of the bioethanol pathways, Pathways I and II have significant WTP GHG emissions, due to high fertilizer usage, particularly nitrogen fertilizer usage, for feedstock production.

Some biofuel LCAs include estimates of land-use change (LUC) GHG emissions. LUC occurs when land is converted to biofuel feedstock production from other uses or states, including non-feedstock agricultural lands, forests, and grasslands. This type of LUC is sometimes called direct LUC. Further, land-use patterns may shift domestically and abroad as markets adjust to changes in crop production levels. This latter type of LUC is called indirect LUC and can be estimated through the use of economic models. LUC can have environmental effects, including changes in soil organic carbon (SOC) that causes carbon emissions or sequestration. We considered that SOC changes might accompany land transitions to sorghum domestically. SOC changes are influenced by soil type, climate, and historical land use [21]. A review of the literature revealed limited and conflicting reports on whether land transitions to sorghum production increases or decreases SOC. For example, Govaerts et al. report results from studies investigating SOC changes at a 30 cm depth in various sorghum cropping systems [22]. Transitioning from continuous sorghum to a sorghum-soybean rotation decreased SOC in five studies. Four studies examining the transition from continuous soybean to a soybean-sorghum rotation, however, were evenly split between exhibiting SOC decreases and increases. In the case of a soybean-sorghum rotation as compared to a continuous soybean, four studies were evenly split between demonstrating SOC increases and decreases. Varvel et al. show a continuous decline in total soil carbon over nearly 20 years of continuous sorghum in research plots in Nebraska [23]. Conversely, Meki et al. produced results from modeling with the Erosion Productivity Impact Calculator that show some tillage practices in sorghum agriculture can increase soil carbon over a 20-year simulation of sorghum fields in 28 Texas counties [24]. Given the spatial and temporal variability in SOC under sorghum, in this analysis, we assumed domestic SOC changes from sorghum production are negligible. As more data become available regarding SOC changes from land transitions to sorghum, we will assess whether sufficient certainty exists to include SOC changes in sorghum ethanol LCA.

In their analysis of GS ethanol, the EPA used economic models to estimate domestic and international LUC associated with GS production. All international LUC is indirect. Domestic LUC includes both direct and indirect LUC. Their analysis accounts for changes in harvested areas, exports, and animal feed use and assumes perfect substitution between GS and corn in the domestic animal feed market [18]. Using a dataset of carbon emission factors for domestic and international land transitions, they estimated total LUC GHG emissions from GS ethanol production of 26 g CO_2_e/MJ. This result exceeds the estimate of LUC GHG emissions associated with corn ethanol that we estimate elsewhere [25]. The EPA has not yet estimated LUC GHG emissions associated with SS or FS ethanol.

Certainly, LUC GHG emissions associated with bioethanol pathways are very uncertain [25,26] because of uncertainty in economic modeling parameters and in the carbon content of affected lands. In the case of sorghum ethanol, this uncertainty could be reduced, if the land use patterns and SOC impacts of sorghum agriculture become better understood.

Relative to the mean GHG emissions from gasoline, sorghum-based ethanol can reduce WTW GHG emissions by 72% for Pathway III(b), as shown in Table 2. For GS-based ethanol, our Pathway I result (with wet DGS as the co-product) shows a life-cycle GHG emission reduction of 35%, or 7% if the EPA’s estimate of LUC GHG emissions (26 g CO_2_e/MJ) is included, as illustrated by Figure 3. Our results differ from the EPA’s primarily because our analysis included a ratio of harvested GS hectares to planted hectares (0.83), which leads to greater fertilizer use. The EPA’s analysis did not consider this ratio. If dried DGS is the co-product, WTW GHG emissions reductions without LUC GHG emissions drop to 23%. Our mean result for Pathway II, which uses RNG from AD of animal waste as the conversion process fuel and produces wet DGS as the co-product, is 56%, if LUC GHG emissions are excluded. Including them decreases the GHG reduction to 29%. If wet DGS is dried, the WTW GHG emission reductions with and without LUC GHG emissions decrease to 27% and 55%, respectively. The EPA’s results for this case were 53% or 52% for wet or dried DGS as the co-product, respectively [18]. Our slightly higher reduction for Pathway II is because we adopted different CHP system parameters than EPA. As a result, in our modeling the conversion step consumes only CHP-produced electricity whereas it consumes some grid electricity in EPA’s analysis [18].

We quantify the uncertainties associated with the WTW GHG emission reductions for each pathway by conducting the Monte Carlo-based stochastic simulations, as shown in Figure 3. Large uncertainties are observed for Pathways I and II because the N_2_O conversion rate of nitrogen fertilizers is uncertain and varies with soil physical properties, climate conditions, and cropland management. Moreover, nitrogen fertilizer usage for GS farming is variable. In fact, it is slightly possible that Pathway I is more GHG emission-intensive than baseline gasoline. On the contrary, the WTW GHG emissions of SS- and FS-based pathways are much less uncertain, partly due to lower nitrogen fertilizer application rates per liter of ethanol produced (see Table 4). Variability in N_2_O conversion rates of nitrogen fertilizers, in levels of enzyme and yeast use, and in the ethanol yields of sugar-based and cellulosic ethanol production are the primary contributors to the observed uncertainty of the SS- and FS-based pathways.

**Table 4:** Probability distribution functions of key parameters of sorghum-based ethanol production pathways

**Parameter**	**Mean**	**P10**	**P90**	**PDF Type**^**g**^
**GS farming**				
Energy use, MJ/kilogram of grain[ [27]	0.68	0.40	0.97	Normal
N, gram/kilogram of grain [9]	24	19	29	Weibull
P_2_O_5_, gram/kilogram of grain [9]	6.4	1.3	12	Logistic
K_2_O, gram/kilogram of grain [9]	0.70	0.16	1.2	Uniform
Grain yield, tonne/hectare [9]	3.4	2.5	4.4	Lognormal
N content of GS stalk, gram/kilogram of grain [28]	10	7.6	11	Triangular
N_2_O conversion rate of N fertilizer:% [20]	1.5	0.41	3.0	Weibull
**SS farming**				
Energy use, MJ/wet tonne of SS [20]	100	90.4	110	Normal
N, gram/wet kilogram of SS [29]	1.5	1.1	1.8	Lognormal
P_2_O_5_, gram/wet kilogram of SS [29]	0.56	0.37	0.76	Normal
K_2_O, gram/wet kilogram of SS [29]	0.89	0.58	1.0	Weibull
Herbicide, gram/wet kilogram of SS [29]	0.069	0.058	0.080	Lognormal
Biomass yield, wet tonne/hectare [29]	76	58	95	Uniform
Grain yield, wet tonne/hectare [30]	1.8	1.0	2.6	Normal
Sugar yield, tonne/hectare [29]	7.0	4.9	9.4	Lognormal
Bagasse yield, wet tonne/hectare [29]	12	8.7	15	Gamma
**FS farming**				
Energy use, MJ/wet tonne of FS^a^	113	102	124	Normal
N, gram/wet kilogram of FS [29,31]	2.2	1.2	3.2	Logistic
P_2_O_5_, gram/wet kilogram of FS [29]	0.41	0.34	0.49	Uniform
K_2_O, gram/wet kilogram of FS [29]	0.82	0.67	0.96	Uniform
Herbicide, gram/wet kilogram of FS [29]	0.067	0.056	0.079	Uniform
FS dry matter yield, tonne/hectare [9]	23	11	36	Weibull
**Ethanol Production**				
**Grain-based ethanol production**				
Ethanol plant energy use, MJ/liter of ethanol^b^[20]	8.1	6.7	9.5	Normal
Ethanol plant energy use, MJ/liter of ethanol^c^[19]	5.1	4.2	6.0	Normal
Ethanol plant energy use, MJ/liter of ethanol^d^	8.3	6.9	9.8	Normal
Ethanol plant energy use, MJ/liter of ethanol^e^	5.3	4.4	6.2	Normal
Ethanol production yield, liter/kilogram of grain [31-35]	0.42	0.40	0.44	Normal
DDGS yield, kilogram /liter of ethanol [20]	0.68	0.61	0.74	Triangular
WDGS yield, kilogram /liter of ethanol [20]	1.9	1.7	2.1	Triangular
Enzyme use, kilogram/tonne of grain [20]	1.0	0.94	1.2	Normal
Yeast use, kilogram/tonne of grain [20]	0.36	0.32	0.40	Normal
**Sugar-based ethanol production**				
Ethanol plant energy use, MJ/liter of ethanol [36]	9.2	9.0	9.3	Uniform
Electricity demand of ethanol production, MJ/liter of ethanol^f^	1.4	1.3	1.5	Uniform
Ethanol production yield, liter/kilogram of sugar [29,31,32,35,37-44]	0.58	0.53	0.62	Lognormal
Yeast use, kilogram/tonne of sugar [42-45]	5.2	4.2	6.2	Uniform
**Cellulosic ethanol production**				
Ethanol production yield, liters/dry kilogram of bagasse [20]	0.38	0.33	0.42	Normal
Enzyme use, kilogram/dry tonne of bagasse [46]	16	9.6	23	Triangular
Yeast use, kilogram/dry tonne of bagasse [46]	2.5	2.2	2.7	Triangular

^a^ Scaled based on yield of FS and SS to the SS farming energy use;

^b^ For FNG-fueled ethanol plants, producing DDGS as the co-product;

^c^ For FNG-fueled ethanol plants, producing WDGS as the co-product;

^d^ For RNG-fueled ethanol plants, producing DDGS as the co-product;

^e^ For RNG-fueled ethanol plants, producing WDGS as the co-product;

^f^ Based on correspondence with Prof. Jaoquim Seabra;

^g^ We employed Easyfit^TM^, a curve-fitting toolbox [47], to find the probability distribution type from a pool of 55 distributions, e.g. Normal distribution, Weibull distribution, Uniform distributions, etc., that best fits each set of the data points we collected for each parameter. For many parameters, we also applied a weighting factor to fit the distribution. The higher the value of the weighting factor corresponding to a sample value of the parameter, the higher possibility the parameter has the sample value in the probability distribution function to be fitted for the parameter. The toolbox uses one of the four well-known methods to estimate distribution parameters based on available sample data: maximum likelihood estimates; least squares estimates; method of moments; and method of L-moments. The toolbox calculates the goodness-of-fit statistics including the Kolmogorov Smirnov statistic, the Anderson Darling Statistic, and the Chi-squared statistic, for each of the fitted distributions. Then, the toolbox ranks the distributions based on the goodness-of-fit statistics. We then selected the distribution with the highest rank primarily based on the Kolmogorov Smirnov statistic. The curve-fitting requires at least five data points for each parameter. We collected sufficient data for the parameters in Table 4 to meet this criterion, except for N content of GS stalk, herbicide use for FS farming, and electricity demand of ethanol production, which we were able to collect only two or three data points. Accordingly, we assumed a uniform or triangular distribution for these parameters.

**Figure 4:**
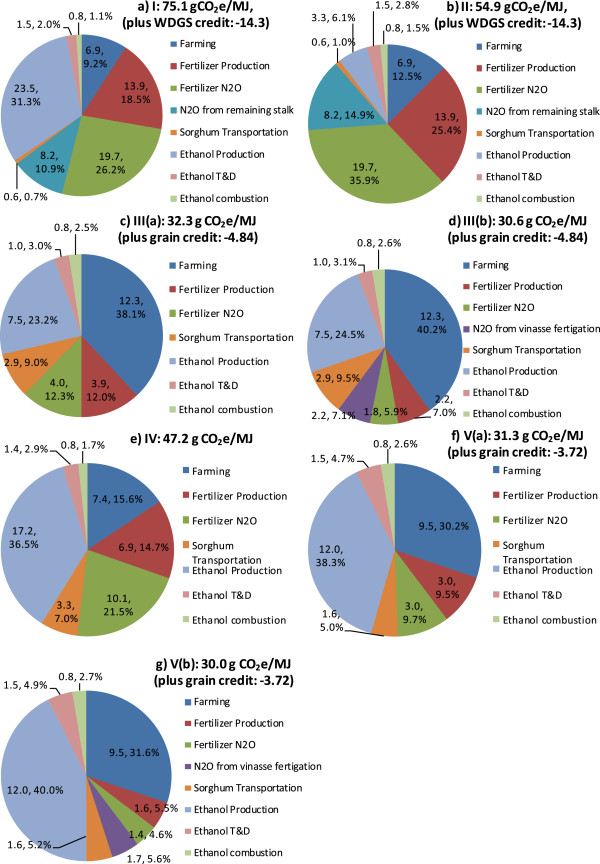
**Contribution (expressed in both g CO**_**2**_**e/MJ and a percentage separated by a comma) of life-cycle activities to well-to-wheels GHG emissions (g CO**_**2**_**e/MJ) of the sorghum-based ethanol pathways.**

The pie charts in Figure 4 show contributions of key life-cycle stages to WTW GHG emissions for the sorghum-based ethanol pathways. For Pathway I, ethanol production is the primary source of the total GHG emissions, accounting for 40% of the total. Fertilizer production and N_2_O emissions from nitrogen fertilizer contribute at the same level. N_2_O emissions from nitrogen in decomposed GS stalks in the field account for 11%; sorghum farming energy use accounts for 9%; and transportation activities account for a minimum share. Pathway II differs from Pathway I only in the ethanol production contribution, with about 20 g CO_2_e/MJ lower emissions from ethanol production. For Pathway III(a), farming energy use is the greatest contributor to life-cycle GHG emissions, followed by ethanol production, N_2_O emissions from nitrogen fertilizer and fertilizer production. Transportation of sorghum feedstock accounts for 9%. Compared to Pathway III(a), the overall WTW GHG emissions of Pathway III(b) are lower and the relative share of fertilizer production and N_2_O emissions from nitrogen fertilizer decreases to 13%, owing to vinasse fertigation and the corresponding reduction in nitrogen fertilizer use. Although only internally-produced energy is consumed, ethanol production still dominates the life-cycle emissions of Pathway IV, due to consumption of sulfuric acid and ammonia in the pretreatment step. Fertilizer production and on-farm N_2_O emissions are responsible for 36% of GHG emissions. Feedstock farming and transportation account for 23%. Pathways V(a) and V(b) do not consume external energy and have fewer emissions from the ethanol production stage, compared to Pathway IV, owing to the integration of cellulosic ethanol production with sugar-based ethanol production, which consumes less process inputs than cellulosic ethanol production. Farming energy use is another major contributor to the WTW GHG emissions, responsible for 30% for Pathways V(a) and 32% for Pathway V(b), respectively; fertilizer production and N_2_O emissions from nitrogen fertilizer account for 19% and 10%, respectively; and transportation activities account for 10% for both cases. For all pathways, ethanol transportation and distribution (T&D) accounted for less than 2 g CO_2_e/MJ.

### Sensitivity analysis of key WTW parameters

**Figure 5:**
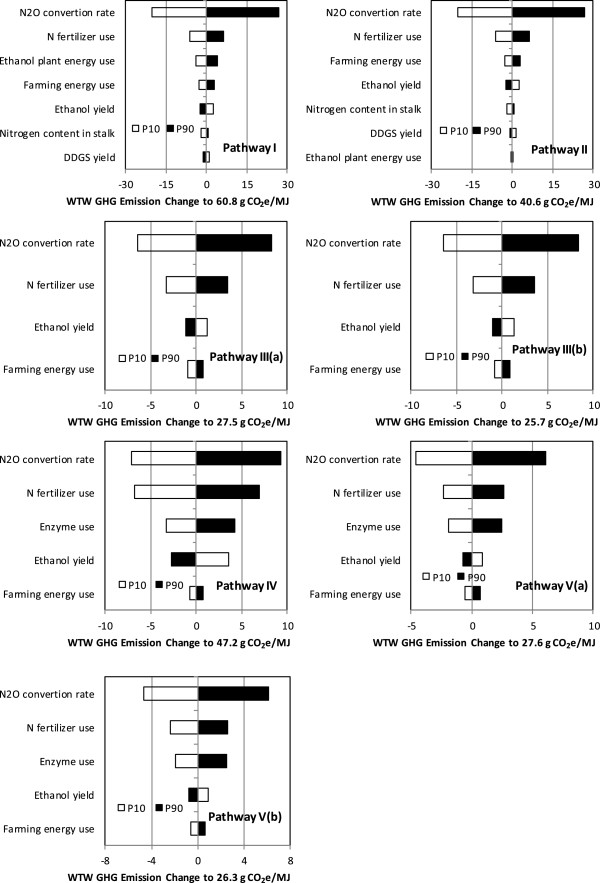
Sensitivity analysis for sorghum-based ethanol pathways.

To investigate the key parameters affecting WTW results of each sorghum-based bioethanol pathway, we used the P10 and P90 values of key parameters in Table 4 to conduct sensitivity analyses. As shown in Figure 5, the N_2_O conversion rate of nitrogen inputs to sorghum fields is the most important factor that influences the WTW results for all cases. Thus, a better understanding of the fertilizer-induced N_2_O emission factor in response to the physical properties of soil and climate that are specific to sorghum fields would improve WTW GHG emissions estimates. In addition, nitrogen fertilizer usage intensity, farming energy use, and ethanol yield are other major factors influencing the WTW results for GS-, SS- and FS-based ethanol. For FS-based cellulosic ethanol production, enzyme use is a more significant factor (Figure 5e) than GS-based ethanol, because in the former case, converting the cellulosic feedstock requires a significantly higher enzyme dosage. Results for corn ethanol and switchgrass ethanol show similar trends [20].

## Discussion

GS ethanol and corn ethanol are similar in that the conversion technology and ethanol yields are nearly identical. Yet, we estimate higher WTW GHG emissions for GS ethanol than for corn ethanol [20]. A key reason for the higher emissions is that sorghum farming is less efficient than corn farming. This may be related to some fundamental constraints to sorghum production, like growth on arid land, less favorable climate, poorer wind and pest resistance than corn. As mentioned earlier, the ratio of harvested to planted hectares for GS (0.83) is lower than this ratio for corn (0.92) [9]. Consequently, the fertilizer intensity and resulting N_2_O emissions per unit of crop harvested are higher for GS. Additionally, N_2_O emissions from decomposed residual GS stalks are higher than N_2_O emissions from residual corn stover because the latter has a lower nitrogen content.

Despite LCA of the total energy balance and economic profitability of SS ethanol in Zimbabwe [48], to our knowledge, there are no LCAs of the fossil energy use and GHG emissions of SS ethanol in the literature to serve as a point of comparison with our results. We therefore compare our SS ethanol results with Brazilian sugarcane ethanol results [20], because both pathways use sugar juice as the feedstock for ethanol production. Our results, with 27.5 and 25.7 g CO_2_e/MJ, for Pathways III(a) and III(b), respectively, are lower than the 45 g CO_2_e/MJ for sugarcane ethanol. SS ethanol has about 12 g CO_2_e/MJ higher GHG emissions associated with feedstock farming and ethanol production than does sugarcane ethanol. Brazilian sugarcane ethanol (as we assumed for use in the US), however, has transportation and distribution emissions 10 g CO_2_e/MJ higher than domestically produced SS ethanol. Furthermore, we do not attribute any LUC GHG emissions to SS ethanol, whereas our analysis of sugarcane ethanol used a value of 16 g CO_2_e/MJ based on literature estimates. The final reason for lower GHG emissions for SS ethanol is our treatment of the grain portion of SS as a co-product that can displace corn. This assumption results in a 4.8 g CO_2_e/MJ (or 5.2% more WTW GHG emission reduction relative to gasoline) credit for SS ethanol.

Our WTW GHG emissions for FS-based cellulosic ethanol using the energy-based allocation method to handle the electricity credit are higher and much more conservative than that when the displacement method is used [20]. Using the displacement method, FS-based cellulosic ethanol has WTW GHG emissions of 34.7 g CO_2_e/MJ, which translates into a reduction of 63% relative to gasoline. We compared our results using the displacement method for the FS pathway to those for other cellulosic ethanol pathways in our previous work [20]. Our results for FS-based cellulosic ethanol are 30, 23 and 42 g CO_2_e/MJ higher WTW GHG emissions than for corn stover-, switchgrass- and miscanthus-based ethanol, respectively. Greater emissions for FS ethanol mainly stem from higher fertilizer-related emissions and accounting for sulfuric acid and ammonia in FS pretreatment [49]. These chemicals contribute 4.2 g CO_2_e/MJ to FS ethanol WTW GHG emissions. This contribution may decrease as pretreatment technology advances, or if other pretreatment methods are used. It is important to note that FS has a very high cellulosic feedstock yield. This could result in an increase of SOC content in lands that produce FS because high-yielding crops enhance SOC. That is, high above-ground biomass yield usually yields high below-ground biomass. For example, miscanthus is a high-yielding crop and CENTURY modeling predicts a significant increase in SOC in soils that produce high-yielding miscanthus [21]. Switchgrass, although still a relatively high-yielding crop, is less productive than miscanthus and as a result has a lower (although still enhancing) effect on SOC.

Results for Pathways V(a) and V(b), which produce ethanol from SS juice and bagasse, show substantial fossil energy savings and GHG emissions reductions. Compared to Pathway III, Pathway V has slightly higher WTW fossil energy use and nearly equivalent WTW GHG emissions per liter of ethanol. The ethanol yield, however, doubles per wet tonne of SS feedstock when both bagasse and juice are converted. Such an integrated ethanol plant might have some unique advantages. For example, SS juice supply may not be sufficient for year-round ethanol plant operation. Tapping the cellulosic portion of the feedstock for ethanol production instead of for electricity generation will lower the export electricity but raise ethanol output from the plant without significant increases of energy use and environmental burdens.

In our analysis, we assumed the SS feedstock supplied to the ethanol plant is crushed for juice extraction the same day to keep a low sugar loss rate of 10% for SS. Considering that sugar losses have been reported as 6% in Brazilian sugar cane ethanol plants [50] and 8% in a Zimbabwean SS ethanol plant [48], this assumption is reasonable. Higher sugar loss rates increase the WTW energy use and GHG emissions of SS-based ethanol. For example, the WTW GHG emissions for Pathway III(a) increase by about 8% and 16% for an increased sugar loss rate of 20% and 30%, respectively, which could represent worst case scenarios of extended SS juice extraction and storage over three days or more [30,51]. A better understanding of the impacts of current and future storage techniques on sugar losses would improve future WTW analysis of SS-based ethanol pathways.

Uncertainty associated with the N_2_O conversion rate in all of the sorghum-based ethanol pathways suggests the need for improved, ideally sorghum-specific fertilizer-induced N_2_O emission factors. Sorghum can generally be grown in arid soil, which emits less N_2_O than moist soils.

Sorghum has a high moisture content. We noticed the moisture contents at harvest of SS and FS moderately impact the WTW GHG emission estimations of SS- and FS-based ethanol pathways. In our analysis, we assume the SS moisture content is 72% with corresponding WTW GHG emissions of 25.7 g CO_2_e/MJ. When the SS moisture content at harvest is 50% and 30%, the WTW GHG emissions for Pathway III(a) would be reduced by 1.4 and 1.9 g CO_2_e/MJ, respectively. A better understanding of harvesting practices for these crops would improve estimates of WTW GHG emissions for SS and FS ethanol pathways.

## Conclusions

We expanded GREET to investigate the life-cycle energy use and GHG emissions of ethanol produced from three types of sorghum in the US. The sorghum-based ethanol pathways can achieve substantial fossil energy savings compared to gasoline. GS-based ethanol production using FNG as the process fuel can achieve moderately lower GHG emission reductions relative to baseline conventional gasoline than corn ethanol. SS ethanol achieves about 71-72% WTW GHG emission reductions. FS ethanol has similar WTW fossil energy use and GHG emission reductions to GS ethanol using RNG. Adding sorghum feedstocks to the existing options for ethanol production could help in meeting the requirements for volumes of renewable, advanced and cellulosic bioethanol production in the US required by the EPA’s RFS program.

## Methods

The overall methodology used for WTW analysis of sorghum-based ethanol pathways can be divided into three main steps: definition of the system boundary of our WTW analysis, as shown in Figure 1; data collection and parametric assumptions; and configuration and expansion of sorghum-based ethanol pathways with GREET. We present our data and parametric assumptions below.

### Data and parametric assumptions

#### Biomass yield

Regardless of type, sorghum has three main components: grain, sugar, and bagasse. The whole plant yield and the yields of each of these components are summarized in Table 5 by sorghum type. Logically, GS has the highest grain yield. Notably, SS may produce substantial quantities of grains—anywhere from 5 to 25% of total dry weight at maturity, depending upon variety [5,52,53]. SS has high bagasse yield, which could be a feedstock for cellulosic ethanol production and biopower generation. We used SS yield data from Florida and Louisiana experimental sites [29,54] with comparable yields. These states are probable sites for SS production. FS has a high yield of dry matter cellulosic biomass. The US Department of Agriculture (USDA) reported 23 dry tonnes per hectare in 2011 [9], which in some regions exceeds the yields of switchgrass and miscanthus [55,56].

**Table 5:** Yields of sorghum biomass and components as ethanol production feedstock

	**GS**	**SS**^**a**^[29,54]	**FS**
Biomass yield (fresh tonne/hectare)		76	85^b^
Biomass moisture content (%)		72	73 [31]
Grain yield (tonne/hectare)	3.4 [9]	2.9	
Sugar yield (tonne/hectare)		6.6	
Bagasse yield (dry tonne/hectare)		12	23 [9]

^a^ The yields are based on field experiments in the absence of data from large-scale production in the US;

^b^ Estimated based on the dry matter yield of 23 tonne per hectare and the reported moisture content of 73% for FS.

#### Farming energy use and fertilizer use

In a GS harvest, only the grain portion of the stalk is removed; stalks remain in the field. Based on the farm machinery energy consumption data from 2004 [27] and the 2004 sorghum yield (716 kilograms/hectare) [9], we estimated that the total energy use for the GS harvest was 0.68 MJ/kilogram. Of the total energy consumed, 35.7% is diesel, 18.5% is gasoline, 45.7% is natural gas, and 0.1% is electricity [57]. In the case of SS, yields and harvesting equipment might be similar to those for Brazilian sugarcane (86.7 tonne/ha) [29,58]. We therefore assumed that the energy use for SS farming is the same as that of Brazilian sugarcane farming (10.0 MJ /tonne SS) [20]. Little data exists on the energy required to harvest FS. This crop’s yield, however, is similar to that of SS. We therefore estimated a value for FS harvest energy by multiplying the SS harvest energy by the ratio of the yields of these two crops.

To determine fertilizer and pesticide application amounts associated with sorghum production (Table 6), we first examined USDA data. The agency does not report data for SS production and combines data for GS and FS production. GS, however, is the dominant sorghum type, accounting for about 96% of US sorghum production in 2011, with the remaining being FS [9]. We therefore assumed that the USDA data represent fertilization rates for GS. Fertilizer and pesticide use for GS farming in our analysis were derived from 2011 state-level USDA data [9] as described in Additional file 1. The fertilizer and pesticide application rates for SS and FS production we used are from field experiments [29,31,54].

**Table 6:** Fertilizer and pesticide inputs for GS (grams per kilogram), SS (grams per wet kilogram) and FS (grams per wet kilogram)

	**GS [**[9]**]**	**SS [**[29]**,**[54]**]**	**FS [**[29]**,**[31]**]**
N	24	1.5	2.2
P_2_O_5_	6.4	0.56	0.41
K_2_O	0.70	0.89	0.82
Herbicide	1.1	0.69	0.67
Insecticide	5.9 × 10^-6^	0	0

#### Feedstock losses during transportation and storage

USDA-reported sorghum yields reflect preharvest losses. Our analysis accounts for additional dry matter losses during transportation and storage, which lower the sorghum effective yield. Table 7 shows dry matter loss during road transportation and storage, as well as the estimated ratio of collected and received biomass.

**Table 7:** Dry matter losses of sorghum biomass during transportation and storage

	**Loss Rate (%)**
Dry matter loss during road transportation	2.0
Dry matter loss during storage	0^a^, 2.6^b^
Ratio of collected and received biomass	1.0^a^, 1.1^b^

^a^ For GS;

^b^ For SS and FS [59].

SS is unique in that it must be managed after harvest to avoid rapid juice and fermentable carbohydrate degradation. As much as 20% of the fermentable sugars can be lost in three days after harvest under typical (room temperature) storage conditions [30]. Eiland et al. [51] indicated that chopped material lost about 50% of fermentable sugars after one week of harvest. Several storage methods have been proposed to limit sugar decomposition during storage including ensilage of SS in large, covered bunkers, cool/cold (no freeze) storage, and drying of whole stalks. Alternatively, it is possible to concentrate extracted juice into a stable syrup and use it as a feedstock. None of these options, however, are viable on an industrial scale because they are capital- and energy-intensive [60]. Optimal timing of SS juice extraction and conversion after harvest is essential. In the analysis, we assumed the juice extraction efficiency is about 80% [32,61] and 10% of sugar would be lost after juice extraction due to handling processes and bacterial decomposition.

#### N_2_O emissions from sorghum farming

N_2_O emissions from sorghum farming are generated from nitrification and denitrification of synthetic nitrogen fertilizers in the soil. When the vinasse that is co-produced from SS ethanol production is used as a fertilizer, it is also a source of N_2_O emissions. Furthermore, crop residue left in the fields after GS harvest decomposes and releases N_2_O. We describe the calculation of N_2_O emissions in our analysis in Additional file 1.

#### Transportation of sorghum feedstock

We used GREET (version released in Dec. 2012) default parameters for truck payload and truck fuel economy for transportation of ethanol feedstocks. We calculated the transportation distance from sorghum fields to ethanol plants, as shown in Table 8 and described in Additional file 1.

**Table 8:** One-way transportation distance from sorghum fields to ethanol plants by truck

**Pathway**	**One-way distance (kilometer)**
I	35
II	35
III	25
IV	33
V	18

#### Energy use of grain-, sugar-, and cellulosic-based ethanol plants

Conversion processes for the three sorghum feedstocks are different and vary in their energy demand and potential for incorporation of CHP. We developed and described a three-step procedure to estimate the net energy demand of the processes in Additional file 1. The results of this process are in Table 9. The GS pathway using RNG as process fuel to feed a CHP facility produces sufficient power and steam to meet process energy demands. Given an ethanol plant with a capacity of 70 million gallons a year, the animal waste from about 308, 000 cows daily is required to produce enough RNG to meet the energy demand of the plant. Therefore, the use of RNG is subject to the availability of manure from farms, and FNG is likely needed in addition to the RNG for the year-around operation of the ethanol plant. The WTW results of both FNG-based and RNG-based pathways provide the opportunity to calculate the weighted averaged life-cycle energy use and GHG emissions for a hybrid production of GS ethanol that consumes both FNG and RNG. Otherwise, the GS pathway has no residual biomass available at the conversion facility to use as a CHP feedstock and consumes a significant amount of fossil energy. In contrast, when SS is the feedstock, combustion of the bagasse provides sufficient heat and power for the process; no external energy is required. With the application of a CHP system and sacrifice of part of the biomass feedstock for steam and electricity generation, the conversion step of the SS- and FS-based pathways achieve 100% energy self-sufficiency.

**Table 9:** Net energy use and electricity credit of each sorghum ethanol pathway

**Pathway**	**Use of CHP system**	**External NG required, MJ/liter**	**External RNG required, MJ/liter**	**External electricity required, MJ/liter**	**Electricity credit, MJ/liter**	**Net energy use, MJ/liter**
I	No	4.4	0	0.70	0	5.1
II	Yes	0	5.3	0	0	5.3
III(a) and (b)	Yes	0	0	0	12.0	0
IV	Yes	0	0	0	2.0	0
V(a) and V(b)	Yes	0	0	0	1.1	0

#### Ethanol yield

We estimated ethanol yields for each type of sorghum. For GS, we adopted an ethanol yield of 0.42 liter/kilogram, based on laboratory experiments reported in the literature [31-35]. The ethanol yield when corn is the feedstock is the same (0.42 liter/kilogram [20]) because corn and GS have comparable starch contents (Additional file 1: Table S1).

In the case of SS, we estimated an average ethanol yield of 0.58 liters/kilogram of sugar for sugar-based ethanol production based on literature accounts of laboratory-scale studies [29,31,32,35,37-44]. This conversion rate is very comparable to that of sugarcane ethanol at 0.58 liters/kilogram sugar from sugarcane [58]. For SS bagasse and FS ethanol, we consider it feasible to achieve 0.38 liters/dry kilogram cellulosic ethanol yield [20].

#### Enzyme and yeast use

Enzyme and yeast consumption levels in grain-based ethanol production were assumed to match their consumption levels for corn ethanol production [20]. The yeast dosage for sugar-based ethanol production was estimated at 5.2 grams /kg substrate according to data from laboratory-scale studies [42-45]. The enzyme and yeast dosages for cellulosic ethanol production from FS were based on cellulosic ethanol production [46]. Energy use and emissions associated with enzyme and yeast production were estimated with GREET [46].

#### Ethanol yields and treatment of co-products

The types of co-products that accompany sorghum ethanol production depend on the feedstock. Grain-based dry-mill ethanol plants co-produce distillers grains with solubles (DGS). DGS can displace conventional animal feeds (including corn, soybean meal, and urea) in beef, dairy, swine, and poultry farms. The grain properties of corn and GS are similar, as are ethanol yields from corn and GS. We therefore assumed the DGS yield in GS ethanol production matched that from corn ethanol production: 1.9 kilograms of wet DGS and 0.68 kilograms of dry DGS per liter EtOH [19,20]. Of the several co-product treatment methods available in GREET, we applied the displacement method [62,63].

SS pathways may produce electricity and vinasse as co-products. In Pathways III and V, we treated the grain SS contains (see Table 5) as a saleable animal feed that displaces corn. The excess electricity that is exported to the grid accounts for about 37% and 5% of the total energy output for Pathways III and V, respectively. For these pathways, we applied energy-based allocation, since both electricity and ethanol are energy products.

The FS-to-ethanol pathway (IV) also produces electricity as a co-product; electricity represents 9% of the total energy output of the ethanol plant. Energy-based allocation was applied to co-products in Pathway IV.

#### Stochastic analysis

Probability distribution functions (PDFs) are developed for key parameters in the sorghum ethanol pathways, as shown in Table 4, for Monte Carlo-based stochastic analysis. The P10 and P90 values represent the 10th and 90th percentiles, respectively, of the distributions of these parameters.

## Competing interests

The authors declare that they have no competing financial interests.

## Authors’ contributions

HC conducted the analysis and writing for this paper with substantial collaboration with JBD, ZW, MQW, and JH. JBD and MQW guided the analysis and writing. ZW collected sorghum farming data. JH provided technical support to GREET configurations and expansion of the sorghum-based pathways. All authors approved the final manuscript.

## Supplementary Material

Additional file 1 Supporting information. Click here for file
