# Supplementary material for: Life-cycle energy use and greenhouse gas emissions of production of bioethanol from sorghum in the United States
Source: Biotechnol Biofuels. 2013 Oct 2;6:141. doi: 10.1186/1754-6834-6-141 (PMC3850671; doi:10.1186/1754-6834-6-141)
Supplement: Additional file 1 — Supporting information. [file 1754-6834-6-141-S1.pdf]

Life-Cycle Energy Use and Greenhouse Gas Emissions of Production of Bioethanol from Sorghum in the United States

Supporting Information

1. Fertilizer application rates for grain sorghum

Fertilizer rates for grain sorghum (GS) were calculated from state-level 2011 U.S. Department of Agriculture (USDA) data with Equation S1.

$$NR = \frac{NR_{AR} \times AR\%}{Area_{harvested} / Area_{planted}} \quad \text{(S1)}$$

Where: NR_{AR} is the nitrogen (N) application rate, in grams per tonne; $AR\%$ is the ratio of planted area receiving N fertilizer; $Area_{harvested} / Area_{planted}$ is the ratio of the harvested area to planted area; and NR is the actual N application rate, in grams per tonne.

The ratio of $Area_{harvested} / Area_{planted}$ adopted (82.7%) is the average of this ratio from 2000 to 2010 [1]. We excluded data from 2011, a severe drought year with a low ratio of 28.7%, from the average. On the other hand, the fertilizer and pesticide use per tonne of sweet sorghum (SS) and forage sorghum (FS) are determined based on a small-scale field experiment of SS and FS farming [2, 3]. The USDA does not maintain data on the production of these two sorghum types. The fertilizer application rates for SS, which are lower than those of GS, agree with previous studies [4, 5]. In addition, the upstream energy use and emissions for fertilizer and chemical

production are simulated separately in the GREET^{TM} (Greenhouse gases, Regulated Emissions, and Energy use in Transportation) model.

2. N_2O emissions

Nitrogen fertilizer application, sorghum residue decomposition, and fertigation of vinasse result in N_2O emissions for GS, SS, and FS farming, as estimated by Equations S2a, S2b, and S2c.

$$E_{GS,N2O} = EF_{N2O,N-Fertilizer} \times Q_{N,Fertilizer} + EF_{N2O,stalk} \times Q_{N,stalk} \quad \text{(S2a)}$$

$$E_{SS,N2O} = EF_{N2O,N-Fertilizer} \times Q_{N,Fertilizer} + EF_{N2O,Vinasse} \times Q_{N,Vinasse} \quad \text{(S2b)}$$

$$E_{FS,N2O} = EF_{N2O,N-Fertilizer} \times Q_{N,Fertilizer} \quad \text{(S2c)}$$

Where: E_{GS,N_2O}, E_{SS,N_2O}, and E_{FS,N_2O} are the N_2O emissions, in grams per liter of ethanol, from GS, SS, and FS fields, respectively;

$EF_{N_2O,N-Fertilizer}$, $EF_{N2O,stalk}$ and $EF_{N2O,Vinasse}$ are the N_2O conversion rates, in %, from synthetic nitrogen fertilizer application, decomposition of sorghum stalk, and vinasse fertigation, respectively;

And $Q_{N,Fertilizer}$, $Q_{N,stalk}$, and $Q_{N,Vinasse}$ are the quantity of nitrogen, in grams, in nitrogen fertilizer, sorghum stalk, and vinasse, respectively.

The same N_2O conversion factor, which is 1.525% [6], is used for nitrogen fertilizer, sorghum stalks, and the vinasse, according to the IPCC Guidelines for National Greenhouse Gas Inventories [7]. N_2O emissions from decomposition of the remaining grain sorghum stalks are

estimated based on the stalk nitrogen content [8], which is 10,000 grams per tonne of grain harvested, and the amount of stalk left. We estimate the N_2O emissions from fertigation of vinasse based on its composition. Vinasse contains 0.63 gram nitrogen per liter ethanol, 1.95 grams potash per liter ethanol, and 0.13 gram potassium per liter ethanol [9].

3. Sorghum feedstock transportation

We estimated the distance of sorghum feedstock transportation based on a supply-demand equilibrium-based radius solution, as shown by Equation S3. Fifty percent of the sorghum yield is assumed to be supplied to ethanol plants with an average annual ethanol production capacity of 265 million liters for corn ethanol plants in 2012 [10].

$$D_{one-way} = \sqrt{\frac{C \times Ratio_{c/r}}{Y_{EtOH,S_i} \times SF}} \qquad \text{(S3)}$$

Where: $D_{one-way}$ is the one-way transportation distance in kilometers from the sorghum field to the ethanol plant; C is the average capacity in million liters of the ethanol plant in 2012; Y_{EtOH,S_i} is the ethanol yield in liters per hectare with ethanol production pathway *i*; SF is the supply factor representing the ratio of the sorghum field area that is harvested and supplied to ethanol plants to the total harvestable sorghum field area; and $Ratio_{c/r}$ is the ratio of collected sorghum biomass to that received by the ethanol plant, as shown in Table 7.

4. Sorghum ethanol production processes

In this section, we outline the three types of ethanol production processes.

4.1 GS-based ethanol production

Figure S1(a) shows the grain-based dry-mill ethanol production processes. In the dry-milling process, the grain is cleaned by removing the debris and other contaminants and then ground into flour, which is slurried with water. A heat-stable enzyme (α-amylase) is added. This slurry undergoes liquefaction then is cooled to approximately 30°C, and a second enzyme (glucoamylase) is added for the saccharification step prior to the final fermentation, which uses yeast [10]. Next, ethanol is separated via distillation then dehydrated. The co-produced is processed into a livestock feed, the distillate grains with solubles (DGS). The sorghum grain-based dry-mill ethanol yield is similar to that of corn ethanol because the two grains have similar starch content as shown in Table S1.

4.2 SS sugar-based ethanol production

Figure S1(b) shows SS sugar-based ethanol production. SS is washed, chopped, and shredded by a set of mill combinations to extract the sucrose-rich juice. Bagasse is collected and burned in a CHP system to generate sufficient steam and electricity for process demands. Surplus electricity is exported to the grid. The extracted juice is then filtered and evaporated to produce molasses, which is sterilized to remove impurities and is then ready to be fermented into ethanol with the addition of yeast. After the process is complete, ethanol is recovered by distillation and subsequent dehydration.

4.3 FS- and SS bagasse-based ethanol

Figure S1(c) shows FS- and SS bagasse-based ethanol production. FS and SS bagasse that consist of cellulose, hemicellulose, and lignin are milled to reduce particle size and pretreated with chemicals and heat prior to enzyme hydrolysis processing. The subsequent fermentation step yields ethanol at a level that is affected by the composition of the feedstock. With similar cellulose and hemicellulose content of sorghum bagasse, as shown by Table S2, we assumed the same ethanol yield of 0.38 liters/dry kilogram of FS or SS bagasse for FS- and SS bagasse-based cellulosic ethanol production as for corn stover ethanol production [6].

5. Energy consumed during sorghum conversion to ethanol

For processes incorporating CHP, we developed a three-step procedure to estimate the net energy demand of the conversion step for each pathway. First, the steam and electricity demand per liter of ethanol in a scenario without process integration was estimated. Second, the potential steam and electricity supply from the CHP system was estimated. Finally the net electricity steam and electricity demand were determined. If CHP-produced steam did not suffice to meet process demands, we assumed 80% efficient natural gas-fired boilers provided the balance. If additional electricity was needed, it was sourced either from the regional central and southern plains generation mix (because feedstock is likely to be produced in this region) or from the U.S. average generation mix.

We estimated total steam and electricity demand of GS ethanol production using fossil natural gas (FNG) as the process fuel based on a USDA ASPEN model. The model results predict that a grain sorghum ethanol plant uses 96.3% of the thermal process energy of a corn ethanol plant and 99.3% of the electrical energy [11]. Accordingly, we estimate that the total energy use for

grain-based dry-mill GS ethanol production using FNG as the process fuel is 0.24 MJ of FNG per MJ of ethanol (86.4% FNG and 13.6% electricity). These estimates are based on GREET's modeling of corn ethanol production in a dry mill with wet DGS as the co-product. For GS ethanol production using renewable natural gas (RNG) as the process fuel, the RNG-fired CHP consumed 0.25 MJ of RNG per MJ of ethanol to provide sufficient steam and electricity at a power-to-steam ratio of 0.12 with a total efficiency of 79.6%, to meet the process energy demand. This configuration of the RNG-fired CHP is feasible [12].

In the case of SS conversion to ethanol, steam demand was based on the unit-level steam demand of a Brazilian sugar and an ethanol co-production plant using sugarcane as the feedstock [13]. The steam demand was allocated between ethanol and sugar by mass (in Brazilian sugar mills, both ethanol and sugar are produced). The result is 0.29 MJ of steam per MJ of ethanol produced. We assume the steam is produced by NG boilers with an efficiency of 80%, and therefore 0.37 MJ of FNG per MJ of ethanol is required. Based on our personal correspondence with Prof. Jaoquim Seabra, the electricity demand averaged 0.066 MJ per MJ of ethanol. Therefore, the total energy use of sugar-based ethanol production is 0.43 MJ per MJ of ethanol.

We assumed conversion of FS in Pathway IV and SS bagasse to ethanol in Pathway V would resemble the production of cellulosic ethanol from corn stover as modeled by Humbird *et al.* [14]. This assumption is reasonable because corn stover has a similar composition to FS and sorghum bagasse (Table S3). Humbird *et al.* [14] estimated a total energy consumption of 0.83 MJ/MJ ethanol, with 0.66 and 0.17 MJ/MJ ethanol for steam demand and electricity demand, respectively.

Bagasse or lignin can be used as a feedstock for CHP in Pathways III, IV, and V. We estimate the steam and electricity generation by CHP based on the amount of biomass available for combustion. The lower heating values (LHV) of the feedstocks were also needed in these calculations. The LHV of SS bagasse is 16.8 GJ/tonne [15]; for lignin, it is 17.9 GJ/tonne [16]. CHP system parameters used were an 80% boiler efficiency for steam generation, an electricity generation efficiency of 20.8% [17], and a waste heat recovery of 65% [18].

For Pathways IV and V, enough feedstock is diverted to the CHP system such that process steam and electricity demand can be met without purchase of supplemental energy. This approach reduces fossil energy consumption and associated greenhouse gas (GHG) emissions. In this case, we estimated the mass fractions of forage sorghum biomass and sweet sorghum bagasse that must be diverted to CHP as 31% and 38%, respectively. Accordingly, an electricity surplus of about 0.56 kWh/liter and 0.32 kWh/liter for Pathways IV and V, respectively, is co-produced.

The default external electricity consumed for sorghum ethanol production is assumed to be supplied by the regional electricity generation mix in the central and southern plains, which consists of 50.5% from coal, 35.7% from FNG, 10.0% from nuclear, 0.2% from biomass, 0.3% from hydropower, and 3.2% from other renewable sources [19]. We also analyze each scenario with the average US grid.

6. WTW results of total energy use, petroleum use, fossil natural gas use, and coal use

Figure S2 shows the WTW total energy use, petroleum use, fossil natural gas use, and coal use of sorghum-based ethanol, in comparison to gasoline.

References

1. **NASS Quick Stats 2.0** [http://quickstats.nass.usda.gov]
2. Tew TL, Cobill RM, Richard EP: **Evaluation of Sweet Sorghum and Sorghum x Sudangrass Hybrids as Feedstocks for Ethanol Production**. *Bioenerg Res* 2008, **1**(2):147-152.
3. Propheter JL, Staggenborg S: **Performance of Annual and Perennial Biofuel Crops: Nutrient Removal during the First Two Years**. *Agron J* 2010, **102**(2):798-805.
4. Lueschen WE, Putnam DH, Kanne BK, Hoverstad TR: **Agronomic Practices for Production of Ethanol from Sweet Sorghum**. *J Prod Agric* 1991, **4**(4):619-625.
5. Wiedenfeld RP: **Nutrient-Requirements and Use Efficiency by Sweet Sorghum**. *Energ Agr* 1984, **3**(1):49-59.
6. Wang M, Han J, Dunn JB, Cai H, Elgowainy A: **Well-to-wheels energy use and greenhouse gas emissions of ethanol from corn, sugarcane and cellulosic biomass for US use**. *Environ Res Lett* 2012, **7**(4).
7. **Guidelines for National Greenhouse Gas Inventories, Volume 4 Agriculture, Forestryand Other Land Use Chapter 11: N2O Emissions from Managed Soils, and CO2 Emissions from Lime and Urea Application** [http://www.ipcc-nggip.iges.or.jp/public/2006gl/pdf/4_Volume4/V4_11_Ch11_N2O&CO2.pdf]
8. Mahama GY: **Variation among grain sorghum genotypes in response to nitrogen fertilizer**. *PhD Thesis*. Kansas State University, Department of Agronomy; 2012.
9. Wilkie AC, Riedesel KJ, Owens JM: **Stillage characterization and anaerobic treatment of ethanol stillage from conventional and cellulosic feedstocks**. *Biomass Bioenerg* 2000, **19**(2):63-102.
10. **Biorefinery Locations** [http://www.ethanolrfa.org/bio-refinery-locations]
11. U.S. Environmental Protection Agency: **EPA sorghum pathway (Air Docket EPA-HQ-OAR-2011-0542, Summary of Modeling Inputs and Assumptions for the Notice of Data Availability (NODA) Concerning Renewable Fuels Produced from GS Under the Renewable Fuel Standard Program)**. 2013.
12. **Catalog of CHP Technologies** [http://www.epa.gov/chp/documents/catalog_chptech_full.pdf]
13. Ensinas AV, Modesto M, Nebra SA, Serra L: **Reduction of irreversibility generation in sugar and ethanol production from sugarcane**. *Energy* 2009, **34**(5):680-688.
14. Humbird D, Davis R, Tao L, Kinchin C, Hsu D, Aden A, Schoen P, Lukas J, Olthof B, Worley M Sexton D, Dudgeon D: *Process Design and Economics for Biochemical Conversion of Lignocellulosic Biomass to Ethanol*. Golden, CO; 2011, Report TP-5100-47764.
15. **Phyllis2 Database** [www.ecn.nl/phyllis2]
16. Blunk SL, Jenkins BM, Kadam KL: **Combustion properties of lignin residue from lignocellulose fermentation**. *Biomass: A Growth Opportunity in Green Energy and Value-Added Products, Vols 1 and 2*. Pergamon: Oxford; 1999:1385-1391.
17. Cai H, Wang M, Elgowainy A, Han J: **Updated greenhouse gas and criteria air pollutant emission factors and their probability distribution functions for electric generating units**. Argonne, IL: Argonne National Laboratory; 2012, Report ANL/ESD/12-2.

18. Frank ED, Han J, Palou-Rivera I, Elgowainy A, Wang M: **Life-cycle analysis of algal lipid fuels with the GREET model**. Argonne, IL: Argonne National Laboratory; 2011, Report ANL/ESD/11-5.
19. **The Emissions & Generation Resource Integrated Database** [www.epa.gov/egrid]
20. **Sorghum** [http://www.starch.dk/isi/starch/sorghum.asp]
21. Patzek TW: **A statistical analysis of the theoretical yield of ethanol from corn starch**. *Natural Resources Research* 2004, **15**(3):205-212.
22. Yan SP, Wu XR, Bean SR, Pedersen JF, Tesso T, Chen YHR, Wang DH: **Evaluation of Waxy Grain Sorghum for Ethanol Production**. *Cereal Chem* 2011, **88**(6):589-595.
23. White PJ, Johnson LA: **Corn Chemistry and Technology (2nd Edition)**. *American Association of Cereal Chemists* 2003.
24. Ai YF, Medic J, Jiang HX, Wang DH, Jane JL: **Starch Characterization and Ethanol Production of Sorghum**. *J Agr Food Chem* 2011, **59**(13):7385-7392.
25. Zhao R, Bean SR, Crozier-Dodson BA, Fung DYC, Wang DH: **Application of acetate buffer in pH adjustment of sorghum mash and its influence on fuel ethanol fermentation**. *J Ind Microbiol Biot* 2009, **36**(1):75-85.
26. Alvarez MM, Perez-Carrillo E, Serna-Saldivar SO: **Effect of decortication and protease treatment on the kinetics of liquefaction, saccharification, and ethanol production from sorghum**. *J Chem Technol Biot* 2010, **85**(8):1122-1129.
27. **The Economic Feasibility of Ethanol Production from Sugar in the United States** [www.usda.gov/oce/reports/energy/EthanolSugarFeasibilityReport3.pdf]
28. Murray SC, Sharma A, Rooney WL, Klein PE, Mullet JE, Mitchell SE, Kresovich S: **Genetic Improvement of Sorghum as a Biofuel Feedstock: I. QTL for Stem Sugar and Grain Nonstructural Carbohydrates**. *Crop Sci* 2008, **48**(6):2165-2179.
29. Wang D, Bean S, McLaren J, Seib P, Madl R, Tuinstra M, Shi Y, Lenz M, Wu X, Zhao R: **Grain sorghum is a viable feedstock for ethanol production**. *J Ind Microbiol Biot* 2008, **35**(5):313-320.
30. Kim M, Day DF: **Composition of sugar cane, energy cane, and sweet sorghum suitable for ethanol production at Louisiana sugar mills**. *J Ind Microbiol Biot* 2011, **38**(7):803-807.
31. Han KJ, Pitman WD, Kim M, Day DF, Alison MW, McCormick ME, Aita G: **Ethanol production potential of SS assessed using forage fiber analysis procedures**. *Gcb Bioenergy* 2012.
32. Mok WSL, Antal MJ: **Uncatalyzed Solvolysis of Whole Biomass Hemicellulose by Hot Compressed Liquid Water**. *Ind Eng Chem Res* 1992, **31**(4):1157-1161.
33. Cherney JH, Lowenberg-DeBoer J, Johnson KD, Volenec JJ: **Evaluation of grasses and legumes as energy resources**. *Proceedings Energy from Biomass and Wastes XII* 1988.
34. Leemhuis RJ, Jong RM: **Biomassa: biochemische samenstelling en conversiemethoden (in Dutch)**. *ECN 72072-GR 2* 1997.
35. Evans RJ, Knight AR, Onischak M, Babu SP: **Development of biomass gasification to produce substitute fuels**. Richland, Washington: Pacific Northwest Laboratory; 1988, Report PNL-6518.
36. Demirbas A: **Calculation of higher heating values of biomass fuels**. *Fuel* 1997, **76**(5):431-434.
37. **Substrate Composition Table** [http://compost.css.cornell.edu/calc/lignin.noframes.html]

38. Lynd LR, Wyman CE, Gerngross TU: **Biocommodity engineering**. *Biotechnol Progr* 1999, **15**(5):777-793.
39. Lee J: **Biological conversion of lignocellulosic biomass to ethanol**. *J Biotechnol* 1997, **56**(1):1-24.
40. Ackerson MD, Clausen EC, Gaddy JL: *Production of ethanol from the lignocellulosic fraction of MSW. Biomass for Energy and Industry: Proceedings of the 7th European Conference*; London. 1994.

Figure S1 Sorghum (a) grain-, (b) sugar-, and (c) cellulosic-based ethanol production processes.

Figure S2 WTW total energy use, petroleum use, fossil natural gas use, and coal use of sorghum-based ethanol.

Table S1 Comparison of the starch content (%) of sorghum grain and corn grain

	Starch Content (%)	Reference
Sorghum grains	74.5	[20]
	65.4–76.3	[21]
	68.7–70.6	[22]
	73	[23]
	72	[23]
	73.24	[24]
	75.8	[24]
	68-70	[25]
	67	[26]
	65	[26]
	68	[26]
	64–74	[27]
Corn grains	69.1–73.6	[28]
	66	[29]
	67.4	[22]

Table S2 Comparison of chemical compositions of sorghum bagasse and corn stover

	Cellulose (%)	Hemicellulose (%)	Lignin (%)	Reference
Sorghum bagasse	45	27	21	[30]
	31	24		[31]
	32	26		[31]
	33	27		[31]
	34	28		[31]
	36	18	16	[32]
	29	27	6	[33]
	33	18	15	[34]
Corn stover	37	25	17	[35]
	33	24	14	[35]
	51	31	14	[36]
	36	29	11	[37]
	36	23	17	[38]
	39	19	15	[39]
	37	28	10	[40]

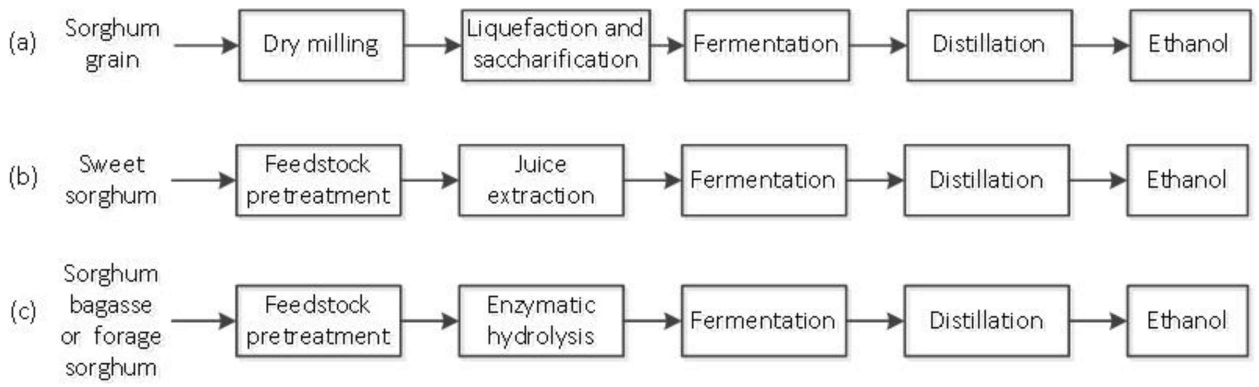

Figure S1 Sorghum (a) grain-, (b) sugar-, and (c) cellulosic-based ethanol production processes.

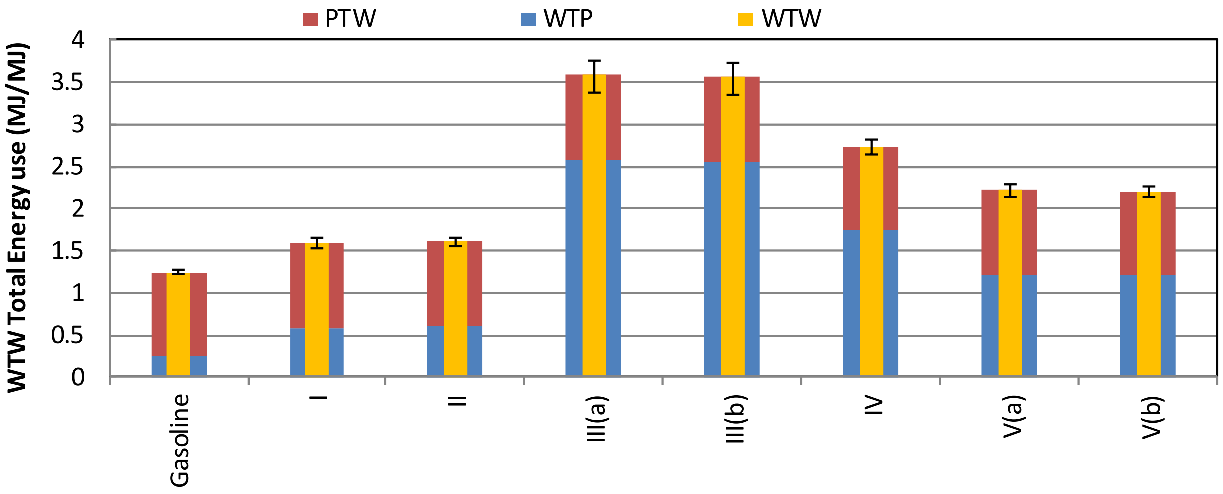
PTW
WTP
WTW
WTW Total Energy use (MJ/MJ)
4
3.5
3
2.5
2
1.5
1
0.5
0
Gasoline
I
II
III(a)
III(b)
IV
V(a)
V(b)

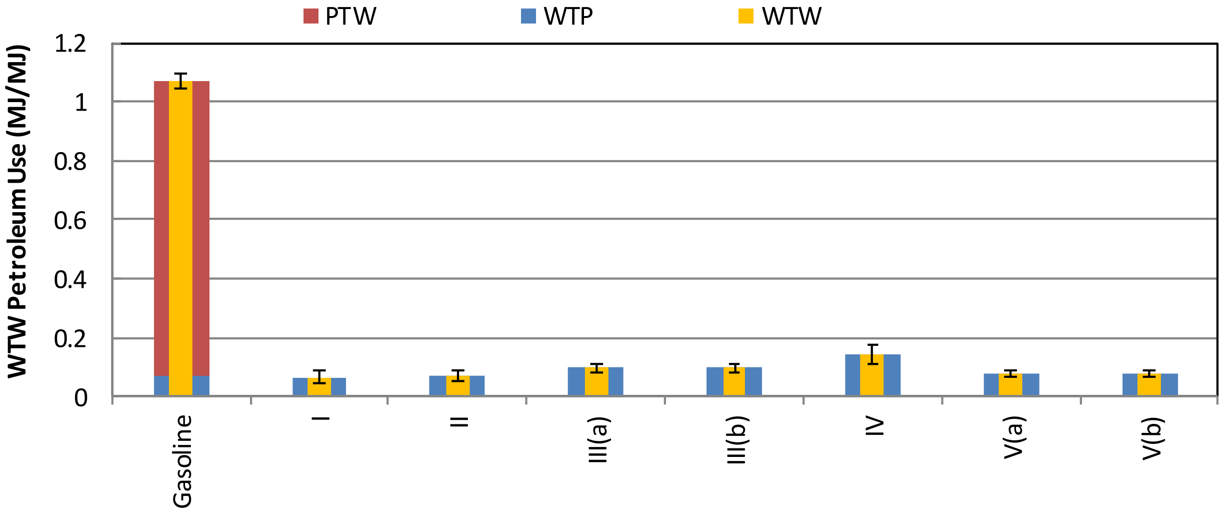
PTW
WTP
WTW
WTW Petroleum Use (MJ/MJ)
1.2
1
0.8
0.6
0.4
0.2
0
Gasoline
I
II
III(a)
III(b)
IV
V(a)
V(b)

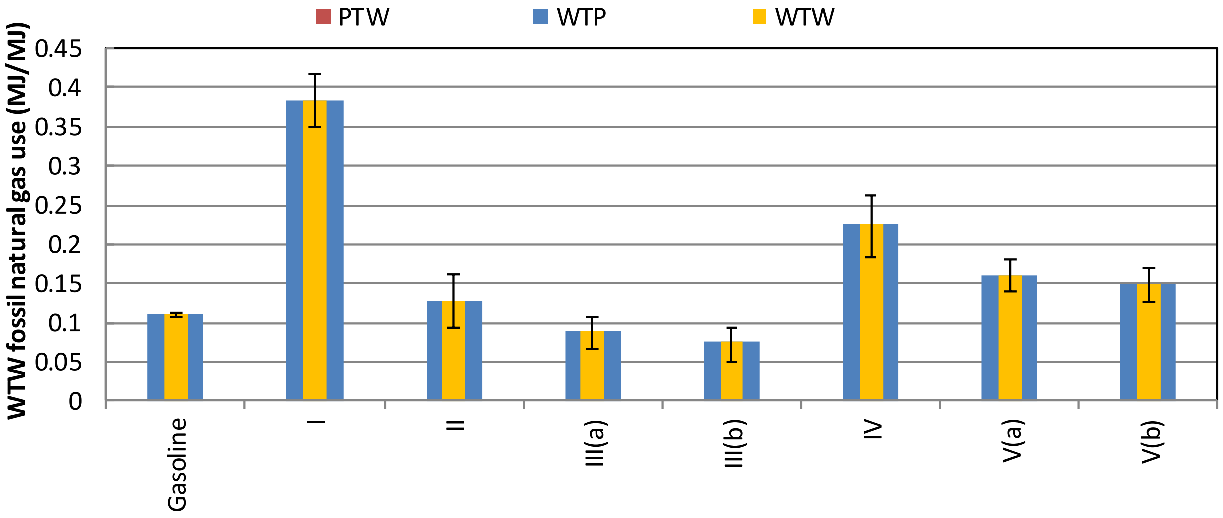
PTW
WTP
WTW
WTW fossil natural gas use (MJ/MJ)
0.45
0.4
0.35
0.3
0.25
0.2
0.15
0.1
0.05
0
Gasoline
I
II
III(a)
III(b)
IV
V(a)
V(b)

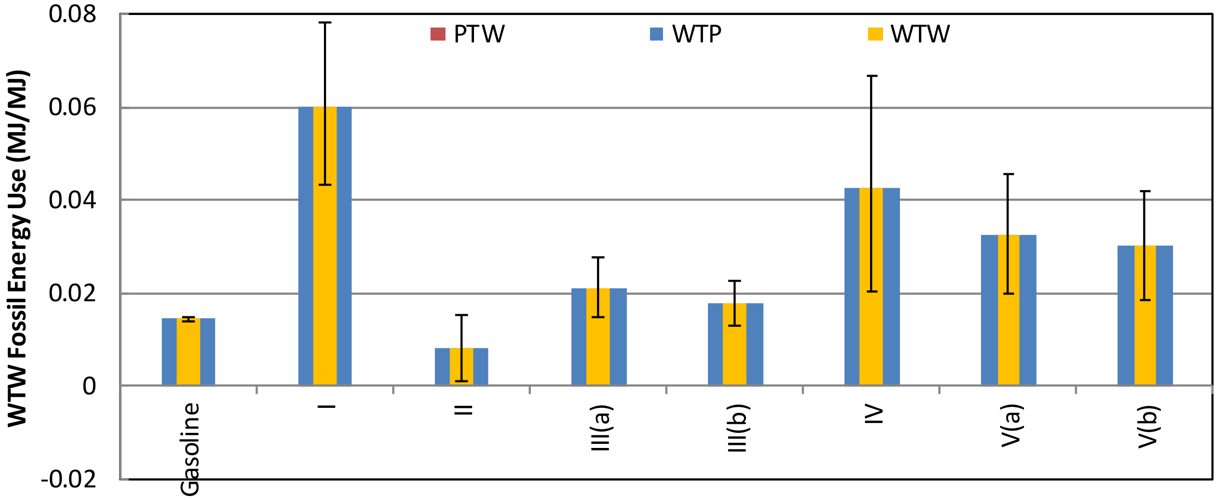

Figure S2 WTW total energy use, petroleum use, fossil natural gas use, and coal use of sorghum-based ethanol.
